# Assessing the Likelihood of Buying Thai Local Snacks

**DOI:** 10.3390/foods13213419

**Published:** 2024-10-27

**Authors:** Wuthiya Saraithong, Kanokwan Chancharoenchai, Nattanicha Chairassamee

**Affiliations:** Faculty of Economics, Kasetsart University, Bangkok 10900, Thailand; wuthiya.s@ku.th (W.S.); nattanicha.chai@ku.th (N.C.)

**Keywords:** consumer behavior, safety, standards, behavioral economics, snacks, buying preference

## Abstract

The snack market in Thailand is growing fast, and yet it faces challenges from the growth in health-conscious consumption. Under these circumstances, it can be quite complicated for people involved in the snack-value chain to respond adequately to more sophisticated demands for snacks. Therefore, this study investigates Thai consumers’ behavior with regard to snacks and its determinants. For the data used in this study, we surveyed 1077 respondents using a questionnaire. Based on a five-point scale, the questions covered respondents’ snack consumption behavior and revealed their preference for additional payment regarding three aspects of snacks: safety and standards, quality, and carcinogen-free snacks. According to an ordered logistic regression, the results show that people’s awareness of food safety and nutrition, and their health consciousness, increase their willingness to pay extra for better-quality snacks. Advertisements and people’s recognition of input sources could also play an essential role in influencing consumers’ preferences. These findings should provide insights for policymakers and producers to catch up with the new demand trend in the Thai snack market.

## 1. Introduction

While there are various types of snack products available, the market is largely dominated by salty snacks, such as chips, popcorn, and pretzels. The snacks on the market often have high levels of saturated fat, salt, and refined sugar, which potentially cause adverse health effects [1]. Given the continuous expansion in the commercial snack market, snack food safety, standards, and quality have increasingly become important issues for consumers and regulatory agencies. Recently, these issues have drawn attention in the international market, resulting in more weight being put on trade policy in the form of trade-related measures. The rising question of how consumers take food safety, standards, and quality into account when making a purchase has captured research interest. A more detailed understanding of consumers’ behavior would equip policymakers and entrepreneurs to formulate appropriate strategies.

**Figure 1:**
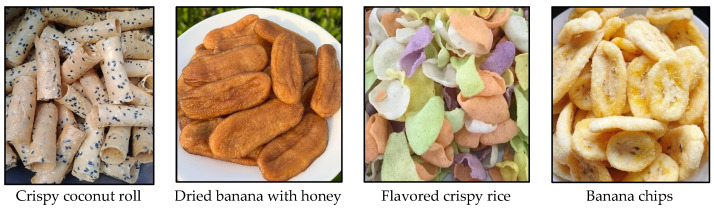
Traditional Thai snacks.

Thailand’s snack market has grown consistently, with a total market value of THB 45,338 million in 2021 (USD 1 = THB 33.53, as of 8 October 2024), an increase of 7.1% from the previous year [2]. There are wide varieties of snacks available in Thailand, ranging from traditional Thai snacks to modern counterparts, influenced by either new consumption trends or foreign culture. Both groups have their own market segments and share some overlapping demand. The main ingredients of conventional Thai snacks usually include local fruits, nuts, rice flour, and sugar. These snacks are, for example, crispy coconut roll, dried banana, sweet dried coconut, banana chips, peanut brittle, and flavored crispy rice, as shown in Figure 1. However, in recent years, foreign-style snacks such as potato chips, pretzels, crispy seaweed, and rice cakes have increasingly gained popularity in the domestic market. They are either wholly imported products or made from imported raw materials. Some raw materials have been proven to contain carcinogenic substances. The regular consumption of contaminated snacks can increase the risk of cancer, creating financial burden for the government.

Apart from the hazardous contaminant issues, healthy snacks can generally be defined by the nutrient balance concept, with limited consumption of sodium, sugar, and fat while maintaining vitamins, minerals, fiber, and protein [3]. Bucher et al. [4] also found that the amount of sugar, fruit, nut, and total fat in the products can be reliable predictors for healthy snacks. People believe that healthy snacks are good for health [5]. The current demand for snacks in Thailand reflects the ongoing global concern for health and food safety, indicating that Thai consumers have generally become more health-conscious. Their concerns for healthy snack products reflect their overall buying behavior and preferences, which are becoming more sophisticated.

Theoretically, people’s consumption behavior depends on their personal traits, both physically and emotionally. In addition, social, economic, political, and cultural factors can affect buyers’ decisions. With the rising importance of the notion of food safety and novel food, several studies looked into this matter, including Hartmann et al. [6], Nørgaard et al. [7,8], Damen et al. [9], Muhammad et al. [10], Hou et al. [11], Ali and Ali [12], and Lysák et al. [13].

Various works, such as Hartmann et al. [6] and Norgaard et al. [7,8], have examined the possible factors that could result in individuals buying food with healthier features. They provided evidence of the significant explanatory power of socioeconomic and related factors for consumers’ behavior and preferences. Hartmann et al. [6] studied how a products’ brand and price stimulated children to choose healthier snacks. Their data were drawn from 116 children in Boston, from 8 to 11 years old, using a questionnaire and choice experiment that centered on a product’s type, brand, and price. From the mixed logit estimation, product type was the most crucial determinant of respondents’ behavior, whereas brand and product price seemed less significant. In contrast, recently, Damen et al. [9] conducted an empirical work on mothers’ buying decisions for snacks for children aged 2–7 years in Italy. Their findings indicated that mothers’ buying decisions varied according to their location. Mothers in the North were more health-conscious, while those in the South were more brand-conscious. Norgaard et al. [7,8] studied Danish adolescents by investigating the factors explaining their preferences and buying intentions regarding novel healthy snacks. From their factor analysis and multiple regression, among such factors as price consciousness, health consciousness, peer influence, social activities, and word of mouth, their findings showed the significance of social factors as the determinant of novel snack preference.

More studies focusing on people’s consumption behaviors regarding healthier food options include the work of Hou et al. [11], which found that consumers favor quality assurance, such as product inspection, more than product traceability, for food safety. Their purchasing decisions were determined by personal characteristics, concerns about food safety, and confidence in food-safety labeling. Information labeling determines consumers’ buying decisions in other cases as well. In the case of different salt-reduction methods for processed potato products, studies have established a strong labeling effect [14]. An additional study on the factors affecting consumers’ buying of health and wellness food products showed the significance of their income and education, based on the Poisson count regression model.

Health consciousness is another key factor, followed by product quality, taste, packaging, and price [12]. Harkness and Areal [15] studied people’s preference for baby food with a reduced level of carcinogens in the United Kingdom. They explored such attributes as packaging, production method, carcinogen level, sugar level, and price. Their estimation based on a mixed logit model showed that, among those attributes, consumers preferred baby food containing less carcinogens.

Another important aspect regarding food standards, safety, and quality is organic food. The factors determining consumers’ decision to buy certified organic food were analyzed using a regression model. The results showed that age, nationality, education, household size, and income were deciding factors for consumers to buy organic food [10]. Lysák et al. [13] investigated the factors influencing consumers buying products made of breadfruit. People who received descriptive information about the products had a higher level of acceptance and were more prepared to buy breadfruit, compared with consumers who had not been so informed. Notably, improvements in related technologies have also stimulated innovation in the food sector. McFadden and Huffman [16] analyzed how consumers responded to a newly developed product. They found that with the availability of food labeling and proper information, people were content to buy newly invented biotech potato products instead of those that had been traditionally produced.

In this paper, we focus on factors likely to affect or influence consumers’ buying decision of snacks with safer and higher quality. These factors include people’s awareness of nutrition issues and food safety, the impact of advertising, and the sources of inputs. This will provide an addition to existing works on consumers’ preference for more healthy food products. The results can help address the sophisticated demand for healthier food products of the more complex world community.

Development in both the demand and supply sides of the snack market has been quite clear. Consumers generally demand safer and healthier snack products. At the same time, technological advances are allowing producers to supply better-quality products. Buyers’ perceptions of snacks’ attributes, such as safety and quality, seem to play a big role in consumption decisions. For example, in Thailand, because people consider locally sourced snacks to be of low quality and inferior to imported products, they prefer them less when purchasing [17].

Anything that could help shape persons’ perceptions or opinions on these matters, then, should affect their purchasing decisions. Therefore, examining factors forming consumers’ perceptions regarding food safety and quality should allow stakeholders to better understand people’s snack preferences in Thailand. This evidence could help generate marketing opportunities for healthier snacks, which currently are not widely available in the mainstream market. As mentioned earlier, conventional snacks can seem less healthy to consumers. However, for safer and higher quality snacks to have a real chance in the market, customers’ behaviors regarding safety, standards, and quality need to be addressed. Therefore, this study’s research question is what the important characteristics may be for improving the marketing opportunities for safer and higher-quality snacks in Thailand. Addressing this issue contributes to the literature on economic behavior, food safety and standards, and business policy, with a special emphasis on the case of snack products in Thailand.

The current study addresses whether Thai consumers are willing to pay extra for safe, good quality, carcinogen-free snacks, and what the key sources of their preference-determined additional payment may be. Thus, this study aims to assess how individual dispositions, such as health concerns, perceptions, and socioeconomic characteristics, explain Thai consumer preferences regarding additional payment for these three aspects. We use statistics and ordered logistic estimations to address these questions.

This paper is organized as follows. Section 2 explains the materials we used and our method. Section 3 reports the findings of this study. Section 4 discusses the main results, and Section 5 concludes.

## 2. Materials and Methods

This section explains the materials and method employed in this study by dividing them into three subsections. The first part describes how the survey was designed. The second part shows the characteristics of the samples, and the last part presents the empirical methodology.

### 2.1. Survey Design

The goal of this research was to assess the probability of changing Thai people’s snack preferences, according to new trends regarding food standards and safety. Relevant information was obtained using questionnaires, which were designed to record respondents’ socioeconomic characteristics and attitudes, as well as to identify their preferences for food standards and safety in snacks. Notably, preference can be an implicit drive of individuals’ behavior. Thus, the self-reported measure was required to evaluate preference based on quantitative values.

This study was carried out under ethical considerations according to the Declaration of Helsinki to protect the research subjects. The Kasetsart University Research Ethics Committee approved the protocol (Certificate of Approval No. COA64/026). The respondents were a purposive sample of people who are Thai citizens aged 13 years or older and have purchased the snacks by themselves. Their snack-buying experiences are required to ensure that they put forward their true preference.

The 1077 volunteers surveyed were asked to complete a questionnaire. The survey was conducted from May to June 2021. Due to the nationwide lockdown as a result of the COVID-19 pandemic, the survey was undertaken through an online channel, using a Google form. In spite of online collection and the random nature of the survey, our sample distribution turned out to be consistent with the country’s demographical structure and covers all regions of Thailand. The data are discussed in detail in Section 2.2.

The survey questionnaire was in the Thai language and had five parts, to elicit individuals’ socioeconomic information, attitudes toward national pride, snack purchasing and consumption behavior, preferences in buying snacks, and factors affecting purchasing decisions. Individuals’ concerns about food standards and safety were included, as mentioned by Nørgaard et al. [7,8], Kongstad and Giacalone [14], and Dinushika and De Silva [18].

To capture respondents’ awareness of how they eat and their belief about its effects on health, they were asked to complete three questions regarding food safety and nutrition issues, the sources of raw materials, and the product attributes. Two additional questions about price consciousness and advertising’s influence on buying decisions were included to elicit behavioral biases. To measure respondents’ sentiments about being Thai and related issues, they were asked five questions to express their opinions regarding their self-esteem and patriotism toward Thailand. The questions were designed to help respondents evaluate their preferences in monetary terms. Respondents were asked to self-declare whether or not they agreed with statements related to their purchasing behaviors and whether they were willing to pay a higher price for safer, higher standard, and better-quality snacks.

The individuals surveyed were asked to measure their preferences for the foregoing issues using a five-point Likert scale, ranging from 1 to 5. The more preferable or agreeable choice was assigned higher scale values, with 1 indicating strongly disagree and 5 indicating strongly agree.

Several related works have provided evidence of the explanatory power of demographic information, concerns and beliefs about food (snack) standards, quality, safety, and purchasing behavior. Those aspects were also covered in the questionnaire, since they can reveal consumers’ snack preferences. We carried out a pretesting procedure, surveying 51 respondents, to check the validity and reliability of the questionnaire before its broader distribution. Cronbach’s alpha statistic was adopted with an acceptable level of 0.7, as suggested by Cronbach [19] and Nunnaly [20]. Based on the pretested questionnaire, the overall Cronbach’s alpha coefficient result was 0.833, indicating that it was reliable with consistent responses.

However, according to the questionnaire and sampling design, the present study has data limitations, as follows.

First, since we collected data online, our samples were possibly limited to people who had access to information online and communication devices, such as computers and mobile phones. These people are likely to live in municipal/urban areas, or Bangkok, the capital of Thailand. The results, therefore, cannot be generalized to populations in other areas, such as nonmunicipal or rural areas, where most people lack access to such devices. Similarly, the policies recommended by the present study are appropriate for the people and snack markets in core urban areas.

Second, this study collected data at one point in time, meaning that the dataset was made up of cross-sectional individual data. In addition, given the small number of observations, the results need to come with a caution about a causal relationship, especially when considering policy implications.

Third, the questions applied a five-point Likert scale to capture respondents’ awareness, belief, attitudes, and behaviors. Such questions have drawbacks. A typical Likert-scale question can measure the orders of the responses but cannot compare between responses. Additionally, respondents are required to choose between given options, which may not match their exact responses. Altogether, this may lead to information lost during measurement [21].

### 2.2. Data Description: Socioeconomic Characteristics

Table 1 shows the socioeconomic characteristics of the 1077 respondents. One-quarter of them were from Central Thailand, which is the country’s most densely populated area, accounting for around 40% of the population [22]. Comparing the respondents’ hometown with their current residence, there was evidence of movement from hometowns to Central Thailand, perhaps because the central region includes the capital city of Bangkok and its environs, where major economic activity occurs. Only 31 respondents (2.88%) declared themselves as being an alternative gender. More than one-half of the respondents were female. Around 60% of respondents were adults, aged from 20 to 39 years. Respondents who had received (or were studying for) a bachelor’s or equivalent degree constituted the majority of the surveyed sample. The statistics showed that there were similar numbers of students, those working for the private sector, and those working for the government or a state enterprise, altogether accounting for more than three-quarters of all respondents. The average monthly income of 311 respondents, or 28.88%, was THB 15,001–25,000. More than one-half of respondents had three or four family members, indicating a medium-sized family. The respondents’ frequency of buying snacks was used to directly represent their consumption behavior, with around half usually buying snacks weekly—once a week (30.08%) and twice a week (20.80%).

### 2.3. Empirical Methodology

This study’s dependent variables were respondents’ preferences regarding the safety, standards, and quality of snacks. Respondents were asked to rate their preferences for each aspect based on a five-point scale, where 1 indicated “strongly disagree” to 5 “strongly agree” with the given statement.

Because of the ordered nature of dependent variables, we used the ordered logistic model to assess the variables that could influence the probability of additional payment for safer snacks (WSAF), higher-quality snacks (WQULI), and snacks without carcinogens (WSNAC). Following Greene [23], the empirical model is defined using Equation (1):

(1) $$Y_{i}^{*}=\beta \times X_{i}^{\prime }+\gamma \times Z_{i}^{\prime }+\varepsilon_{i},$$

where $Y_{i}^{*}$ is a variable related to the preferences for snacks as expressed by the intention to pay a higher price by surveyed individual i. Since the study measures three aspects (food safety, standards, and quality), the estimations are separated into three regression equations, in which $X_{i}^{\prime }$ represents the vector of seven sociodemographic variables of surveyed individual i. The rest of the variables are denoted by the vector $Z_{i}^{\prime }$, which includes eight variables related to other types of behavior, perception, and awareness. The descriptions and abbreviations of these relevant variables in the specification are shown in Table 2.

In the above equation, β and γ refer to the corresponding estimated parameters. However, the parameters obtained from regression Equation (1) cannot directly present the effect of those variables on the probability of changes in respondents’ preference levels. To circumvent this limitation, we apply the marginal effect for a particular variable. The statistical test of the hypothesis for the significant explanation of each variable is based on the t-statistic at the conventional level of at least a 10% significance level (α ≤ 0.10) or a 90% confidence level.

## 3. Results

This section is divided into three subsections. The first part provides descriptive statistics of the relevant variables. The second part presents the outcome of the ordered logistic models, and the last part covers the marginal effect analysis.

### 3.1. Descriptive Statistics

The descriptive statistics results are separated into three topics as follows.

#### 3.1.1. Respondents’ Awareness and Beliefs

Based on the five-point Likert scale questions, the results in Table 3 show the average points of five issues related to respondents’ awareness of, concerns, and beliefs about food products, especially snacks. Every issue is presented in detail by the number of respondents choosing each rating scale and the mean value of these scales, as shown in Table 3. Unsurprisingly, price is shown to play a key role in consumers’ buying decisions, at least among the samples, as seen from the highest mean of 4.02 out of 5. The average scores of food safety, standard, and quality, especially the carcinogen-related case, and product attributes are given quite similar rating scores, averaging around 3.7. However, awareness of food safety and nutrition standards was slightly lower, with an average of 3.64, as a result of respondents giving relatively less importance to the appearance of packaging and nutrition issues.

The individuals surveyed also expressed their thoughts on safety, standards, and quality through price and product issues by giving high scores to price, product-related attributes, and clear labeling about ingredients. Overall, the importance of price factors in consumers’ purchasing decisions is quite clear, but their concerns for safety, standards, and quality issues cannot be denied.

Notably, the influence of an advertisement on buying decisions had the lowest mean but the highest unreported-standard deviation compared with the other items. This might indicate the wider dispersion of the confidence in advertising content and its influencing power on buying decisions in certain groups of respondents.

#### 3.1.2. National Sentiment

Table 4 reveals different opinion-levels regarding the national sentiment of the 1077 respondents. The highest average score, of 3.53, is for the opinion that a priority should be given to benefitting the Thai people over others. Even though respondents did not agree that Thailand is the best country (average score 2.86) and a leader in ASEAN (average score 2.34), they still had positive views regarding being Thai (average score 3.10).

Overall, the average sentiment level score for all five issues is only 2.94, slightly less than the moderate level. At least in this study, the surveyed respondents revealed less pride in their nation.

#### 3.1.3. Preference to Pay More for Food Safety and Quality

As shown in Table 5, the respondents seemed highly concerned about these issues, with average scores above 3.76 for all three issues. Most respondents were concerned about food quality when they purchased snacks (average score 3.85). Following the theory of demand, the respondents moderately agreed with the statement of additional payment for safer snacks. However, the average score was the highest (average score is 3.86) when the question related to carcinogen contamination, suggesting a higher preference for paying more for snacks without carcinogens than for generally safer products (average score is 3.55). This confirms that respondents are quite aware about safety, standards, and quality attributes before deciding to purchase snacks. In addition, they accept the trade-off between what they eat and paying a higher price.

### 3.2. Ordered Logistic Regression Results

As explained in the methodology section, we applied the ordered logistic regression of the three aspects of the safety, standards, and quality of snacks to address the explanatory power of relevant variables regarding the probability of respondents’ preference for those three aspects. Based on the principle of threshold values from the ordered logit analysis, there is significant power in the variables to changes in preference levels. Since the preferences are rated on a five-point scale (from 1 = strongly disagree to 5 = strongly agree), the threshold result would be 4. If the results of four thresholds are significant, the independent variables are thus significant, improving the level of preferences, for example, from agree to strongly agree.

As the empirical results show in Table 6, all our threshold values are statistically significant at the 0.01 level. Consequently, all independent variables could improve preference levels in all cases—for safer snacks (WSAF), quality of snacks (WQULI), and snacks without carcinogen (WSNAC).

The findings shown in Table 6 indicate that gender (GENFA), hometown (NE), education (EDU), number of family members (NFAM), being a student (OCST), frequency of purchasing snacks (FREQ), national pride (PRIDE), and family income (FAMI) do not have a significant effect on consumers’ preferences for the specific snack issues.

The overall results indicate what was expected. Awareness of food safety and nutrition issues (BEV), concern about product price (PPRICE), and advertising influence (ADV) could significantly explain the consumption preferences for all three issues of food safety, standards, and quality.

Income (INC) and preference about the sources of input (CASA) strongly support a preference for food safety in the case of snacks (WSAF). At the same time, the age of respondents (AGE) negatively affects WSAF. Notably, the preference for the product attribute regarding the packaging details (PPROD) seems to violate common belief, as indicated by a negative sign.

As mentioned earlier, the estimated parameters cannot directly present how the factors impact the buying-preference scale or how they would affect the chance of increasing the respondents’ preferences from lower to higher scales. Consequently, the marginal effects with respect to particular factors were calculated and are reported in the next section.

### 3.3. Marginal Effects

Table 7 shows the marginal effects of the significant variables on the preference for safer snacks (WSAF) and the full results of all independent variables are provided in Table A1 in Appendix A. We find that older adults are less likely to agree with the statement. A one-year increase in age increases the probability of strongly disagreeing, disagreeing, and being neutral with the statement by 0.4%, 0.9%, and 1.6%, respectively.

Age affects only the additional payment for food safety but not other willingness-to-pay preferences. Our results show that older adults are not willing to pay for safer food. This is supported by the study of Cates et al. [24], which showed that older adults considered themselves knowledgeable about food safety. However, many of them were not following recommended food safety practices. With the optimistic bias of older adults, they also perceived themselves to have lower levels of risk for foodborne illness than other individuals [25]. Altogether, this could explain why older consumers are unwilling to pay for food safety.

In our study, income is also a factor impacting only the preference for safer snacks. As expected, consumers with higher incomes are willing to pay for food safety. There are positively significant marginal effects on agree and strongly agree. An increase in one unit of income results in a 1.2% and 2.0% higher degree in the agree and strongly agree categories, respectively, with the statement. This implies that consumers earning higher incomes consider their safety when purchasing snacks.

Purchasing-related factors, nutrition, and production sources show similar patterns of marginal effects. Consumers who considered these factors before purchasing snacks are more likely to agree and strongly agree with the statement, indicating that they are willing to pay a higher price for safer snacks. Our results show consistency; people concerned about food safety would definitely pay more to prevent them from facing that issue.

A concern for reasonable pricing also positively impacts the preference for safer snacks. If consumers are more concerned about pricing, item quality, and quantity, they are more likely to agree and strongly agree to pay extra for safer snacks.

Advertisements also have an impact on the preference for safer snacks. The results show that consumers receiving information from advertisements about safe snacks are more likely to pay more for safer snacks. The marginal effect of strongly agree with the statement (6.5%) is almost double that for agree (approximately 4%).

Table 8 reports the factors affecting the preference for good-quality snacks (WQULI) and the full results of all independent variables are provided in Table A2 in Appendix A. Overall, the respondents have somewhat extreme opinions regarding the quality issue of snacks, since there was no significant factor for the agree level.

Unsurprisingly, the results indicate that the more consumers are concerned about food nutrition, the higher the probability that they strongly agree with the statement. Consumers who are seriously aware of food nutrition tend to consider food quality when they purchase snacks. On average, a one-point increase in concern about food nutrition enhances by approximately 20% the chance of strongly agreeing with having a higher preference for good-quality snacks.

However, consumers concerned about product elements tend to be less concerned about the quality of snacks. The probability of strongly agreeing with the statement decreases by approximately 4% for consumers who highly consider these product elements. Contrarily, consumers who are concerned about reasonable pricing tend to consider the quality issue in their purchase. An increase in one point for this pricing concern increases the probability of strongly agreeing with a higher preference for good quality snacks by approximately 11%. Our findings indicate that consumers expect the product price to reflect quality. In contrast, consumers concerned less about quality issues are more likely to purchase snacks by considering the packaging, brands, flavors, and other product-related elements.

Consumers receiving information from advertisements about snack quality are also more likely to be concerned about the quality of their purchase. As expected, an advertisement could be a channel to improve consumer perceptions of food safety, standards, and quality, leading to a preference for such products.

The results in Table 9 are similar to those in Table 8, with the full results of all independent variables provided in Table A3 in Appendix A. Respondents have possibly extreme opinions about the non-carcinogen quality of snacks, since there was no significant factor for the agree level.

Consumers concerned about food nutrition and reasonable pricing and receiving information from advertisements have an increased probability of purchasing carcinogen-free snacks by 13.6%, 9.1%, and 11.8%, respectively.

Similarly, an increase in one point of concern about raw material sources leads to an increased probability of 5% of strongly agreeing with the statement of preferring snacks without carcinogen contamination.

However, consumers concerned about product elements, such as packaging, taste, or quantity, are less likely to agree with the statement. The probability of strongly disagreeing, disagreeing, and having a neutral preference for snacks without carcinogens increases by 0.8, 0.8, and 2.3%, respectively. These results could be explained by product-related elements strongly influencing consumers’ snack purchasing decisions. Popular snack brands and other elements that are easily noticed are more likely to be purchased without concern for carcinogen-contamination. This could lead consumers to have a lower preference for carcinogen-free snacks. Not only do entrepreneurs need to take this issue into account in developing a business strategy, but the government also should consider it as a visible cause of health problems and enforce standard regulations for the disclosure of ingredients.

## 4. Discussion

From the results presented, we identify the solid explanatory power of such variables as BEV, ADV, and CASA in explaining consumers’ preferences and, then, their behavior regarding snacks. Individuals concerned about food safety and nutritional issues, especially when purchasing snacks (BEV), are generally likely to prefer safer, higher-quality, and carcinogen-free snacks. These consumers’ preferences translate into their consumption behavior, possibly taking the form of a readiness to pay a higher price for these products. Consumers’ awareness of food safety reflects their health consciousness. The statistical significance of this matter as the determinant of consumers’ buying behaviors is consistent with other studies empirically undertaken in various contexts [11,12,26,27].

Furthermore, Iqbal et al. [28] examined the organic food market and found that people’s health consciousness and safety concerns were positively related to their intention of purchasing organic food products. In addition, investigating organic food, Rana and Paul [29] showed that a person’s consumption attitude was primarily affected by non-infectious causes, such as heart disease and depression. Other studies supporting the relationship between health consciousness and consumers’ buying behaviors include Chancharoenchai’s [30] and Saraithong’s [31], which focused on consumer’ purchasing intentions when there was improvement in product attributes in the cases of fresh milk and beef, respectively.

As explained above, the statistical significance of ADV in all models indicates the importance of advertisements and their impact on consumers’ buying behaviors. An advertisement is likely to motivate persons to favor safer, higher-quality, and carcinogen-free snacks. According to the classification of market structure, the snacks market could be considered monopolistic, where many sellers are operating in the market and selling differentiated products [32]. Because of the large market size and great variety of snack products, an advertisement could provide a channel for manufacturers to communicate with their customers, especially about the specific character of their products. Additionally, an advertisement could psychologically stimulate demand. As shown previously, the shift in consumers’ behavior as the result of an advertisement is consistent with Wang et al. [33], who showed that advertising content can have different impacts on the demand for healthy and unhealthy food and beverage items.

Snacks, with their numerous manufacturers, can be considered a high-competition market. For products to remain competitive in the market, while dealing with the increasing trend of food safety, they must not compromise their tastes and appearances. Snack businesses may have to vigorously carry out product research either by themselves or by collaborating with other institutions. They need to come up with products that abide by food safety but are still tasty and look nice. And in some cases, the tastes and appearances of snacks may need to be adjusted to accommodate the change in consumers’ behavior. At the same time, education and communication with the public about the safety and quality of snacks is also as important. This education can be undertaken by private companies to their target customers or through government channels to wider audiences.

In the case of CASA, as presented earlier, the sources of inputs could also affect consumption behaviors. People aware of the input sources were likely to choose safer and carcinogen-free snacks. This finding is supported by Jensen et al. [34], Memery et al. [35], and Giraud et al. [36], who reported positive relationships between buyers’ decisions and locally produced goods.

These findings emphasize the importance of knowledge and the information provided through advertisements, which offer a channel for market communication and allow consumers to realize product differentiation. Because of the significance of these issues, the government and the private sector should collaborate in formulating strategies to expedite information distribution. With the proper policy implementation, consumers could be encouraged to change their purchasing patterns in a welfare-induced manner.

In this study, we are aware of data and measurement limitations. To improve this research, future studies may consider using a novel fuzzy Likert scale [21] and conducting experiments to correct respondents’ biases during data collection. In addition, collecting data continuously as longitudinal datasets could reveal respondents’ behaviors, attitudes, and beliefs regarding food safety and quality over time. This is appropriate to evaluate policies and plan business strategies.

## 5. Conclusions

This study examines factors that could influence Thai consumers’ preferences and behaviors regarding snacks. It investigates several issues related to food safety, quality, and standards. To achieve its objective, this study was undertaken based on data from a questionnaire survey with 1077 respondents. Generally, the respondents were aware of food safety, nutrition, the quality of input sources, prices, and products. However, they showed little concern about national sentiment, leading to indifference in preference for different product aspects.

The three issues related to consumers’ behavior regarding snacks are estimated separately by three different models using ordered logistic regression. The marginal effects of significant variables are calculated because the regression coefficients could not provide a detailed interpretation. Those marginal effects show the probability of respondents’ changes based on their opinion levels. From the results, a strong explanatory power of independent variables is found for people’s awareness about the safety and nutrition of products and advertisements. To a lesser extent, variables such as national pride, education level, being a woman, and registering as an alternative gender could also determine patterns of snack consumption. Following these findings, policymakers should implement appropriate policies to support and improve the welfare of stakeholders at a micro level and generate growth in the snack industry at the macro level.

## Figures and Tables

**Table 1:** Socioeconomic characteristics and consumption behavior of surveyed respondents.

Characteristics		Number	Percentage
Hometown	North	142	13.18
	Northeast	202	18.76
	West	112	10.40
	Central	275	25.53
	East	142	13.18
	South	204	18.94
Gender	Men	405	37.60
	Female	641	59.52
	Alternative	31	2.88
Age (Years)	13–19	137	12.72
	20–29	352	32.68
	30–39	340	31.57
	40–49	171	15.88
	≥50	77	7.15
Education (Graduated or Enrolled)	Less than a bachelor’s degree or diploma	174	16.16
	Bachelor’s or diploma	673	62.49
	Master’s degree	206	19.13
	Above master’s degree	24	2.23
Occupation	Student	267	24.79
	Private employee	267	24.79
	Government or state enterprise employee	295	27.39
	Business owner	148	13.74
	General employee	60	5.57
	Unemployed	29	2.69
	Retired	11	1.02
Income (THB per month)	≤5000	151	14.02
	5001–15,000	237	22.01
	15,001–25,000	311	28.88
	25,001–35,000	184	17.08
	35,001–45,000	90	8.36
	≥45,000	104	9.66
Family Members	1–2	114	10.58
	3–4	594	55.15
	5–6	336	31.20
	>6	33	3.06
Frequency of Buying Snacks	Every day	157	14.58
	Twice per week	224	20.80
	Once per week	324	30.08
	Once per month	202	18.76
	Uncertain	170	15.78

Note: USD 1 = THB 33.53, as of 8 October 2024.

**Table 2:** Abbreviations and definitions of relevant variables.

Abbreviations	Definitions
Preference for snack purchasing decision ($Y_{i}^{*}$)	
WSAF	Respondents’ opinion on buying preference for safer Thai snacks. The five-point Likert scale indicates the preference on the three issues regarding dependent variables. A higher value on the scale indicates greater support for paying more for safer snacks.
WQULI	Respondents’ opinion on buying preference for quality of Thai snacks.
WSNAC	Respondents’ opinion on buying preference for carcinogen-free Thai snacks
Sociodemographic characteristics ($X_{i}^{\prime }$)	
NE	This variable denoted the respondents’ hometown as a dichotomous value = 1 for respondents whose hometown is in Northeastern Thailand, facing more severe poverty than other regions, otherwise = 0.
GENFA	Respondents’ gender. Female respondents are marked as 1, otherwise = 0.
AGE	Respondents’ age. It is divided into five groups based on generation, as presented in Table 1, marked as the numbers 1–5 from age 13–19 to 50 years old and over.
EDU	Respondents’ education. Educational levels, measuring knowledge and understanding, are marked as follows: 1 = below primary school, 2 = primary school, 3 = secondary school, 4 = high school or equivalent, 5 = bachelor’s degree or equivalent, 6 = master’s degree, and 7 = above master’s degree.
NFAM	The number of family members of respondents, which reflects either purchasing power or health concern. Having five persons or more in the family = 1; otherwise = 0.
OCT	Respondents’ occupation. The occupation variable is grouped into two types: respondents’ occupation is student = 1 and otherwise = 0.
INC	Respondents’ average income per month. It is divided into six levels from least to highest income as shown in Table 1.
Behaviors, perception, and awareness ($Z_{i}^{\prime }$)	
PRIDE	National pride of Thai people is an average score of respondents’ national sentiment.
FREQ	Respondents’ frequency of buying snacks. This variable is grouped according to Table 1 with 1–5 scale.
FAMI	Respondents’ purpose of buying snacks. If the intended consumer of the snacks is themselves and their family, this variable is marked as 1, otherwise = 0.
BEV	It is the average of respondents’ awareness of food safety and nutritional attributes of snacks.
CASA	This variable is the mean of respondents’ awareness of food safety, standards, and quality of snacks.
PPROD	This variable is the average of respondents’ preference for product attributes.
PPRICE	This is based on the average opinion level of related issues to show respondents’ preference for product price.
ADV	This variable indicates the influence of advertising on buying decisions.

**Table 3:** Surveyed respondents’ awareness of and beliefs about selected issues.

Issues	Rating of Awareness and Belief Scale (Least to Most)					Mean
	1	2	3	4	5	
1. Awareness of food safety and nutrition (BEV)						
- Safety issues (expiration date, hygiene, place of purchase, source of raw materials, and no additives)	30 (2.79)	74 (6.87)	285 (26.46)	397 (36.86)	291 (27.02)	3.78
- Appearance of packaging ^a^	33 (3.06)	106 (9.84)	464 (43.08)	354 (32.87)	120 (11.14)	3.39
- Nutrition issues (fat, sodium, and other nutrient contents)	71 (6.59)	113 (10.45)	373 (34.63)	319 (29.62)	201 (18.66)	3.43
Average score for all issues = 3.64						
2. Awareness of food safety, standards, and quality (CASA)						
- Willing to pay more for food that does not contain carcinogens	41 (3.81)	92 (8.54)	222 (20.61)	434 (40.30)	288 (26.74)	3.78
- Willing to pay more if cassava chips are labeled stating that they are made from Thai cassava without carcinogens	48 (4.46)	86 (7.99)	256 (23.77)	424 (39.37)	263 (24.42)	3.71
Average score for all issues = 3.74						
3. Awareness of products (PPROD)						
- Attractive packaging	24 (2.23)	60 (5.57)	454 (42.15)	390 (36.21)	149 (13.83)	3.54
- Clear, detailed labeling of ingredients	10 (0.93)	47 (4.36)	261 (24.23)	433 (40.20)	326 (13.83)	3.95
- Quantity contained in the package	15 (1.39)	33 (3.06)	251 (23.31)	468 (43.45)	310 (28.78)	3.95
- Product name	30 (2.79)	84 (7.80)	435 (40.39)	355 (32.96)	173 (16.06)	3.52
- Various flavors	18 (1.67)	40 (3.71)	261 (24.23)	432 (40.11)	326 (20.27)	3.94
Average score for all issues = 3.78						
4. Awareness of price of products (PPRICE)						
- Appropriate price for the quantity of snacks	9 (0.84)	34 (3.16)	244 (22.66)	439 (40.76)	351 (32.59)	4.01
- Suitable price for snacks’ quality	8 (0.74)	35 (3.25)	215 (19.96)	426 (39.55)	393 (36.49)	4.08
- Reasonable price for products processed locally	10 (0.93)	38 (3.53)	252 (23.40)	449 (41.69)	328 (30.45)	3.97
Average score for all issues = 4.02						
5. Purchasing decisions influenced by advertising media (ADV)	75 (6.96)	137 (12.72)	324 (30.08)	362 (33.61)	179 (16.62)	3.40
Average score for all issues = 3.40						

Notes: Numbers in parentheses are percentages from the total sample of 1077. The mean is the average of the five rating scales for various items related to that issue. ^a^ Appearance of packaging consists of the aspects of products visible to consumers. These include, for example, packaging cleanliness and durability, which constitute the safety of products in general.

**Table 4:** National sentiment of surveyed respondents.

Issue	Rating of Sentiment Form Scale (Least to Most)					Mean
	1	2	3	4	5	
1. Thailand should focus on the benefits of the Thai people first.	46 (4.27)	589 (54.69)	360 (33.43)	50 (4.64)	32 (2.97)	3.53
2. Being Thai is good.	79 (7.34)	323 (29.99)	373 (34.63)	230 (21.36)	72 (6.69)	3.10
3. Overall, Thailand is the best country compared with other countries in the world.	93 (8.64)	205 (19.03)	358 (33.24)	304 (28.23)	117 (10.86)	2.86
4. Cultural influences from other nations have diluted Thai-ness.	80 (7.43)	193 (17.92)	400 (37.14)	301 (27.95)	103 (9.56)	2.86
5. Thailand is an economic and political leader in the ASEAN region.	67 (6.22)	86 (7.99)	284 (26.37)	354 (32.87)	286 (26.56)	2.34
Average score for all issues = 2.94						

Notes: Numbers in parentheses are percentages from the total sample of 1077. The mean is the average of the five rating scales related to that issue.

**Table 5:** Preference to pay more of surveyed respondents.

Item	Rating of Preference Scale (Least to Most)					Mean
	1	2	3	4	5	
1. Preference to pay more for safer snacks	36 (3.34)	98 (9.10)	350 (32.50)	419 (38.90)	174 (16.16)	3.55
2. Impact of quality on snacks purchase decision	32 (2.97)	72 (6.69)	221 (20.52)	452 (41.97)	300 (27.86)	3.85
3. Buying snacks without carcinogens	46 (4.27)	55 (5.11)	227 (21.08)	423 (39.28)	326 (30.27)	3.86
Average score for all issues = 3.76						

Notes: Numbers in parentheses are percentages from the total sample of 1077. The mean is an average of five rating scales for various items related to that issue.

**Table 6:** Results of ordered logistic regression.

Variable	Estimated Parameter		
	WSAF	WQULI	WSNAC
NE	−0.139	−0.112	−0.032
	(0.156)	(0.160)	(0.157)
GENFA	0.026	0.026	−0.146
	(0.132)	(0.129)	(0.127)
Age	−0.168 **	−0.085	−0.074
	(0.073)	(0.073)	(0.074)
EDU	0.102	−0.019	0.116
	(0.087)	(0.086)	(0.085)
NFAM	0.010	−0.070	−0.024
	(0.086)	(0.084)	(0.085)
OCST	0.100	0.314	−0.160
	(0.191)	(0.202)	(0.210)
INC	0.188 ***	0.049	0.055
	(0.054)	(0.052)	(0.055)
PRIDE	0.093	−0.073	0.064
	(0.092)	(0.095)	(0.096)
FREQ	0.031	0.052	0.067
	(0.047)	(0.048)	(0.050)
FAMI	0.138	0.105	0.057
	(0.125)	(0.124)	(0.126)
BEV	0.685 ***	1.262 ***	0.944 ***
	(0.147)	(0.166)	(0.160)
CASA	0.318 ***	0.200	0.351 ***
	(0.108)	(0.124)	(0.124)
PPROD	−0.051	−0.301 **	−0.285 *
	(0.142)	(0.140)	(0.152)
PPRICE	0.526 ***	0.823 ***	0.633 ***
	(0.120)	(0.123)	(0.124)
ADV	0.594 ***	0.558 ***	0.817 ***
	(0.084)	(0.087)	(0.088)
Threshold:			
Y = 2	4.186 ***	4.004 ***	4.886 ***
	(0.636)	(0.649)	(0.640)
Y = 3	6.016 ***	5.792 ***	6.095 ***
	(0.640)	(0.650)	(0.650)
Y = 4	8.431 ***	7.914 ***	8.297 ***
	(0.672)	(0.680)	(0.686)
Y = 5	10.918 ***	10.607 ***	10.797 ***
	(0.704)	(0.722)	(0.721)
Observation	1077	1077	1077
R^2^	0.177	0.227	0.226
Log pseudolikelihood	−1204.763	−1107.568	−1120.228

Notes: Robust standard errors are in parentheses. * *p* < 0.10; ** *p* < 0.05; *** *p* < 0.01.

**Table 7:** Marginal effects on preference for safer snacks (WSAF).

Variables	Strongly Disagree	Disagree	Neutral	Agree	Strongly Agree
Age	0.004 **	0.009 **	0.016 **	−0.011 **	−0.019 **
	(0.002)	(0.004)	(0.007)	(0.005)	(0.008)
INC	−0.005 ***	−0.010 ***	−0.018 ***	0.012 ***	0.021 ***
	(0.002)	(0.003)	(0.005)	(0.004)	(0.006)
BEV	−0.018 ***	−0.037 ***	−0.065 ***	0.044 ***	0.075 ***
	(0.004)	(0.008)	(0.014)	(0.010)	(0.017)
CASA	−0.008 ***	−0.017 ***	−0.030 ***	0.021 ***	0.035 ***
	(0.003)	(0.006)	(0.010)	(0.007)	(0.012)
PPRICE	−0.014 ***	−0.029 ***	−0.050 ***	0.034 ***	0.058 ***
	(0.003)	(0.007)	(0.011)	(0.008)	(0.013)
ADV	−0.016 ***	−0.032 ***	−0.056 ***	0.039 ***	0.065 ***
	(0.003)	(0.005)	(0.007)	(0.006)	(0.009)

Notes: Only significant variables reported in Table 7 are displayed. Full marginal results are in the appendix. Robust standard errors are in parentheses. ** *p* < 0.05; *** *p* < 0.01.

**Table 8:** Marginal effects on preference for good quality snacks (WQULI).

Variables	Strongly Disagree	Disagree	Neutral	Agree	Strongly Agree
BEV	−0.027 ***	−0.049 ***	−0.097 ***	−0.003	0.176 ***
	(0.005)	(0.007)	(0.013)	(0.006)	(0.023)
PPROD	0.006 **	0.012 **	0.023 **	0.001	−0.042 **
	(0.003)	(0.005)	(0.011)	(0.001)	(0.019)
PPRICE	−0.017 ***	−0.032 ***	−0.063 ***	−0.002	0.115 ***
	(0.004)	(0.005)	(0.009)	(0.004)	(0.017)
ADV	−0.012 ***	−0.022 ***	−0.043 ***	−0.002	0.078 ***
	(0.003)	(0.004)	(0.007)	(0.002)	(0.011)

Notes: Only significant variables reported in Table 8 are displayed. Full marginal results are in the appendix. Robust standard errors are in parentheses. ** *p* < 0.05; *** *p* < 0.01.

**Table 9:** Marginal effects on preference for snacks without carcinogen (WSNAC).

Variables	Strongly Disagree	Disagree	Neutral	Agree	Strongly Agree
BEV	−0.028 ***	−0.027 ***	−0.077 ***	−0.004	0.136 ***
	(0.005)	(0.005)	(0.014)	(0.004)	(0.023)
CASA	−0.010 ***	−0.010 ***	−0.029 ***	−0.001	0.051 ***
	(0.004)	(0.004)	(0.010)	(0.002)	(0.018)
PPROD	0.008 *	0.008 *	0.023 *	0.001	−0.041 *
	(0.005)	(0.004)	(0.012)	(0.001)	(0.022)
PPRICE	−0.019 ***	−0.018 ***	−0.052 ***	−0.003	0.091 ***
	(0.004)	(0.004)	(0.010)	(0.003)	(0.018)
ADV	−0.024 ***	−0.023 ***	−0.067 ***	−0.003	0.118 ***
	(0.004)	(0.004)	(0.007)	(0.003)	(0.011)

Notes: Only significant variables reported in Table 9 are displayed. Full marginal results are in the appendix. Robust standard errors are in parentheses. * *p* < 0.10; *** *p* < 0.01.

## Data Availability

The original contributions presented in this study are included in the article. Further inquiries can be directed to the corresponding author.

## Appendix A

Full Results of Marginal Effects.

**Table A1:** Full empirical results of Marginal effects on preference for safer snacks (WSAF).

Variables	Strongly Disagree	Disagree	Neutral	Agree	Strongly Agree
NE	0.004	0.008	0.013	−0.009	−0.015
	(0.004)	(0.008)	(0.015)	(0.010)	(0.017)
GenFA	−0.001	−0.001	−0.002	0.002	0.003
	(0.003)	(0.007)	(0.012)	(0.009)	(0.014)
Age	0.004 **	0.009 **	0.016 **	−0.011 **	−0.019 **
	(0.002)	(0.004)	(0.007)	(0.005)	(0.008)
EDU	−0.003	−0.006	−0.010	0.007	0.011
	(0.002)	(0.005)	(0.008)	(0.006)	(0.010)
NFAM	−0.000	−0.001	−0.001	0.001	0.001
	(0.002)	(0.005)	(0.008)	(0.006)	(0.009)
OCST	−0.003	−0.005	−0.009	0.007	0.011
	(0.005)	(0.010)	(0.018)	(0.012)	(0.021)
INC	−0.005 ***	−0.010 ***	−0.018 ***	0.012 ***	0.021 ***
	(0.002)	(0.003)	(0.005)	(0.004)	(0.006)
PRIDE	−0.002	−0.005	−0.009	0.006	0.010
	(0.002)	(0.005)	(0.009)	(0.006)	(0.010)
FREQ	−0.001	−0.002	−0.003	0.002	0.003
	(0.001)	(0.003)	(0.004)	(0.003)	(0.005)
FAMI	−0.004	−0.008	−0.013	0.009	0.015
	(0.003)	(0.007)	(0.012)	(0.008)	(0.014)
BEV	−0.018 ***	−0.037 ***	−0.065 ***	0.044 ***	0.075 ***
	(0.004)	(0.008)	(0.014)	(0.010)	(0.017)
CASA	−0.008 ***	−0.017 ***	−0.030 ***	0.021 ***	0.035 ***
	(0.003)	(0.006)	(0.010)	(0.007)	(0.012)
PPROD	0.001	0.003	0.005	−0.003	−0.006
	(0.004)	(0.008)	(0.013)	(0.009)	(0.016)
PPRICE	−0.014 ***	−0.029 ***	−0.050 ***	0.034 ***	0.058 ***
	(0.003)	(0.007)	(0.011)	(0.008)	(0.013)
ADV	−0.016 ***	−0.032 ***	−0.056 ***	0.039 ***	0.065 ***
	(0.003)	(0.005)	(0.007)	(0.006)	(0.009)

Notes: Robust standard errors are in parentheses. ** *p* < 0.05; *** *p* < 0.01.

**Table A2:** Full empirical results of Marginal effects on preference for good quality snacks (WQULI).

Variables	Strongly Disagree	Disagree	Neutral	Agree	Strongly Agree
NE	0.002	0.004	0.009	0.000	−0.016
	(0.003)	(0.006)	(0.012)	(0.001)	(0.022)
GenFA	−0.001	−0.001	−0.002	−0.000	0.004
	(0.003)	(0.005)	(0.010)	(0.000)	(0.018)
Age	0.002	0.003	0.007	0.000	−0.012
	(0.002)	(0.003)	(0.006)	(0.000)	(0.010)
EDU	0.000	0.001	0.001	0.000	−0.003
	(0.002)	(0.003)	(0.007)	(0.000)	(0.012)
NFAM	0.001	0.003	0.005	0.000	−0.010
	(0.002)	(0.003)	(0.006)	(0.000)	(0.012)
OCST	−0.007	−0.012	−0.024	−0.001	0.044
	(0.004)	(0.008)	(0.015)	(0.002)	(0.028)
INC	−0.001	−0.002	−0.004	−0.000	0.007
	(0.001)	(0.002)	(0.004)	(0.000)	(0.007)
PRIDE	0.002	0.003	0.006	0.000	−0.010
	(0.002)	(0.004)	(0.007)	(0.000)	(0.013)
FREQ	−0.001	−0.002	−0.004	−0.000	0.007
	(0.001)	(0.002)	(0.004)	(0.000)	(0.007)
FAMI	−0.002	−0.004	−0.008	−0.000	0.015
	(0.003)	(0.005)	(0.010)	(0.001)	(0.017)
BEV	−0.027 ***	−0.049 ***	−0.097 ***	−0.003	0.176 ***
	(0.005)	(0.007)	(0.013)	(0.006)	(0.023)
CASA	−0.004	−0.008	−0.015	−0.001	0.028
	(0.003)	(0.005)	(0.010)	(0.001)	(0.017)
PPROD	0.006 **	0.012 **	0.023 **	0.001	−0.042 **
	(0.003)	(0.005)	(0.011)	(0.001)	(0.019)
PPRICE	−0.017 ***	−0.032 ***	−0.063 ***	−0.002	0.115 ***
	(0.004)	(0.005)	(0.009)	(0.004)	(0.017)
ADV	−0.012 ***	−0.022 ***	−0.043 ***	−0.002	0.078 ***
	(0.003)	(0.004)	(0.007)	(0.002)	(0.011)

Notes: Robust standard errors are in parentheses. ** *p* < 0.05; *** *p* < 0.01.

**Table A3:** Full empirical results of Marginal effects on preference for snacks without carcinogen (WSNAC).

Variables	Strongly Disagree	Disagree	Neutral	Agree	Strongly Agree
NE	0.001	0.001	0.003	0.000	−0.005
	(0.005)	(0.004)	(0.013)	(0.001)	(0.023)
GenFA	0.004	0.004	0.012	0.001	−0.021
	(0.004)	(0.004)	(0.010)	(0.001)	(0.018)
Age	0.002	0.002	0.006	0.000	−0.011
	(0.002)	(0.002)	(0.006)	(0.000)	(0.011)
EDU	−0.003	−0.003	−0.009	−0.000	0.017
	(0.003)	(0.002)	(0.007)	(0.001)	(0.012)
NFAM	0.001	0.001	0.002	0.000	−0.003
	(0.002)	(0.002)	(0.007)	(0.000)	(0.012)
OCST	0.005	0.005	0.013	0.001	−0.023
	(0.006)	(0.006)	(0.017)	(0.001)	(0.030)
INC	−0.002	−0.002	−0.004	−0.000	0.008
	(0.002)	(0.002)	(0.004)	(0.000)	(0.008)
PRIDE	−0.002	−0.002	−0.005	−0.000	0.009
	(0.003)	(0.003)	(0.008)	(0.000)	(0.014)
FREQ	−0.002	−0.002	−0.006	−0.000	0.010
	(0.001)	(0.001)	(0.004)	(0.000)	(0.007)
FAMI	−0.002	−0.002	−0.005	−0.000	−0.008
	(0.004)	(0.004)	(0.010)	(0.001)	(0.018)
BEV	−0.028 ***	−0.027 ***	−0.077 ***	−0.004	0.136 ***
	(0.005)	(0.005)	(0.014)	(0.004)	(0.023)
CASA	−0.010 ***	−0.010 ***	−0.029 ***	−0.001	0.051 ***
	(0.004)	(0.004)	(0.010)	(0.002)	(0.018)
PPROD	0.008 *	0.008 *	0.023 *	0.001	−0.041 *
	(0.005)	(0.004)	(0.012)	(0.001)	(0.022)
PPRICE	−0.019 ***	−0.018 ***	−0.052 ***	−0.003	0.091 ***
	(0.004)	(0.004)	(0.010)	(0.003)	(0.018)
ADV	−0.024 ***	−0.023 ***	−0.067 ***	−0.003	0.118 ***
	(0.004)	(0.004)	(0.007)	(0.003)	(0.011)

Notes: Robust standard errors are in parentheses. * *p* < 0.10; *** *p* < 0.01.
